# Identify Down syndrome transcriptome associations using integrative analysis of microarray database and correlation-interaction network

**DOI:** 10.1186/s40246-018-0133-y

**Published:** 2018-01-19

**Authors:** Min Chen, Jiayan Wang, Yingjun Luo, Kailing Huang, Xiaoshun Shi, Yanhui Liu, Jin Li, Zhengfei Lai, Shuya Xue, Haimei Gao, Allen Chen, Dunjin Chen

**Affiliations:** 1 Department of Fetal Medicine and Prenatal Diagnosis, the Third Affiliated Hospital of Guangzhou Medical University, Guangzhou, 510150 China; 2 Obstetrics and Gynecology Institute of Guangzhou, Guangzhou, 510150 China; 3 The Medical Centre for Critical Pregnant Women in Guangzhou, Guangzhou, 510150 China; 4 Key Laboratory for Major Obstetric Diseases of Guangdong Province, Guangzhou, 510150 China; 5 Key Laboratory for Reproduction and Genetics of Guangdong Higher Education Institutes, Guangzhou, China; 6 Department of Mathematics, University of California, Berkeley, CA 94720 USA; 7 Mendel Genes Inc, Manhattan Beach, CA, Manhattan Beach, CA 90266 USA; 8 Department of Thoracic Surgery, Nanfang Hospital, Southern Medical University, Guangzhou, China; 9 State Key Laboratory of Respiratory Disease, The First Affiliated Hospital of Guangzhou Medical University, National Clinical Research Center for Respiratory Disease, Guangzhou, China

**Keywords:** lncRNA, DSCR9, Down syndrome, Protein–protein interaction, Correlation-interaction-network, Neurological diseases

## Abstract

**Background:**

Long non-coding RNAs (lncRNAs) have previously been emerged as key players in a series of biological processes. Dysregulation of lncRNA is correlated to human diseases including neurological disorders. Here, we developed a multi-step bioinformatics analysis to study the functions of a particular Down syndrome-associated gene DSCR9 including the lncRNAs. The method is named correlation-interaction-network (COIN), based on which a pipeline is implemented. Co-expression gene network analysis and biological network analysis results are presented.

**Methods:**

We identified the regulation function of DSCR9, a lncRNA transcribed from the Down syndrome critical region (DSCR) of chromosome 21, by analyzing its co-expression genes from over 1700 sets and nearly 60,000 public Affymetrix human U133-Plus 2 transcriptional profiling microarrays. After proper evaluations, a threshold is chosen to filter the data and get satisfactory results. Microarray data resource is from EBI database and protein–protein interaction (PPI) network information is incorporated from the most complete network databases. PPI integration strategy guarantees complete information regarding DSCR9. Enrichment analysis is performed to identify significantly correlated pathways.

**Results:**

We found that the most significant pathways associated with the top DSCR9 co-expressed genes were shown to be involved in neuro-active ligand-receptor interaction (GLP1R, HTR4, P2RX2, UCN3, and UTS2R), calcium signaling pathway (CACNA1F, CACNG4, HTR4, P2RX2, and SLC8A3), neuronal system (KCNJ5 and SYN1) by the KEGG, and GO analysis. The A549 and U251 cell lines with stable DSCR9 overexpression were constructed. We validated 10 DSCR9 co-expression genes by qPCR in both cell lines with over 70% accuracy.

**Conclusions:**

DSCR9 was highly correlated with genes that were known as important factors in the developments and functions of nervous system, indicating that DSCR9 may regulate neurological proteins regarding Down syndrome and other neurological-related diseases. The pipeline can be properly adjusted to other applications.

**Electronic supplementary material:**

The online version of this article (10.1186/s40246-018-0133-y) contains supplementary material, which is available to authorized users.

## Introduction

Down syndrome (DS) is the most common chromosome disorder occurring in about one per 700 newborns each year [1]. Although it has been well established that an extra copy of chromosome 21 causes DS, the genetic and molecular mechanisms of the disease are yet unclear. Studies on partial trisomy have led to the characterization of a region of the chromosome 21 known as Down syndrome critical region (DSCR), which is located at the distal end of the long arm of chromosome 21 (21q22.1–22.3) and has candidate genes whose imbalance may induce a marked cognitive deficit as well as other pathologies and associated conditions [2]. Although the involvement of DSCR as the sole cause of DS symptoms is still controversial, previous studies have suggested that this region plays a primary role in the genetic interactions related to the pathogenesis of DS. Nevertheless, it has not been completely understood what exact subset of genes that are over-expressed on chromosome 21 generating these DS-related deficiencies. Most studies focus on protein-coding genes in DSCR, whereas little is known about the three long non-coding RNAs (lncRNAs): DSCR8, DSCR9, and DSCR10.

LncRNAs are a large class of non-protein-coding transcripts that are greater than 200 bases in length and are involved in numerous physiological and pathological processes [3]. Only a small number of lncRNAs have been characterized functionally, while most of them were shown to control gene expression by regulating various aspects of gene expression [4]. Many lncRNAs are shown to regulate important cancer hallmarks including proliferation, apoptosis, metastasis, metabolism, senescence, and drug-resistance [5]. In addition, cumulative evidence have demonstrated that lncRNAs contribute to the complex biological system organization and gene regulatory networks of the central nervous system affecting brain patterning, neural stem cell maintenance, neurogenesis and gliogenesis, stress responses, and synaptic and neural plasticity. A number of lncRNAs are linked to neurological diseases such as the dysregulated BACE1-AS and BC200 in Alzheimer’s disease [6]. However, evidence of any lncRNA being involved in DS has not yet been fully elucidated. Previous studies showed that NRON (ncRNA repressor of the nuclear factor of activated T cells) was an lncRNA mediating the cytoplasmic to nuclear shuttling of the NFAT transcription factor. In animal models, deregulation of the DSCR1 and DYRK1A acts synergistically to prevent nuclear occupancy of NFATc transcription factors leading to reduced NFATc activity and to a number of features of DS [7]. However, a conclusive link between this lncRNA and DS pathophysiology has not been reported so far.

In current study, we aimed to find lncRNAs that are related to Down syndrome by establishing a systematic bioinformatics analysis as well as the pipeline to predict functions of lncRNAs on human chromosome 21 and by validating their potential regulatory target mRNAs by qPCR. After mining the RNA expression data from Affymetrix transcriptional profiling microarrays, the functions of the DSCR9 lncRNA were found to be enhanced in neurological-related pathways, which might cause Down syndrome and other neurological diseases.

## Materials and methods

### lncRNA probe localization

The probe sequence of Affymetrix U133 Plus 2 Platform (http://www.ncbi.nlm.nih.gov/geo/query/acc.cgi?acc=GPL570) containing over 4000 sets of data were obtained from NCBI GEO database. They were aligned with the human genome hg19 and GENCODE (version 18) using the BLAT with parameters ‘-stepSize = 5-repMatch = 1,000,000-minScore = 0-minIdentity = 0’ based on an efficient algorithm for microarray probe re-annotations [8]. BLAT results with no more than two mismatches were saved for our study.

### Expression data collection and pre-processing

The gene expression data from NCBI GEO database that mentioned above were retrieved from EBI ArrayExpress database [9] by Bioconductor package ArrayExpress [10]. EBI, NCBI, and DDBJ are three high-throughput data exchanging portals, where data will be updated to the newest. In particular, the chip data of EBI is extremely clear, which includes details of the data such as sample information, chip location information, chip signals, and so on. So we chose the EBI database and the data therein for our analysis. Incorporated CEL files were pre-processed using robust multichip average (RMA) normalization method. The standard deviation of the expression levels was calculated. LncRNAs including HOTTIP, HOTAIR, and DSCR9 were analyzed. Datasets with low standard deviation level (< 0.25) were filtered out, and the remaining experimental data were used for proceeding the analysis. According to [11], and using 0.25 as the threshold, we find that the transcriptomic changes of the data are significant enough to determine the lncRNA-related genes. Therefore, we utilize this value (0.25) as the cutoff to guarantee high quality of the data and in the meanwhile capture significantly correlated transcriptome information.

### Statistics analysis

Pearson correlation between lncRNA probe (e.g., HOTTIP, HOTAIR, or DSCR9) and other 54,674 probes were calculated. *p* values were presented as unmodified *p* values. Multiple testing corrections were performed using the *q* value package in R [12]. Genes with *q* values lower than 0.05 were considered as significant to be co-expression genes of lncRNA.

### Protein–protein interaction (PPI) network for co-expression genes

The protein–protein interaction (PPI) network, including all the top co-expression genes of DSCR9, was constructed to identify the most important functional relevance of DSCR9. Moreover, all the PPI relationships from the following databases: HPRD, IntAct, MIPS, BIND, DIP, MINT, PDZBase, and Reactome, were combined to gain a more comprehensive understanding of the interactions between DSCR9 and its potential target genes based on a previously described method [13]. We used default options of the abovementioned eight networks and interactomes that were found in at least one of these networks will be integrated into our analysis. In this manner, all relevant interactomes regarding DSCR9 will be considered. Finally, the PPI network was visualized with Cytoscape software [14].

### Biological pathway analysis

Genes that showed the highest correlation with lncRNA expression level were used for the biological pathway analysis. Parameters used in our pathway analysis were listed as following: (1) two pathway databases were included: KEGG pathway database [15] and Reactome pathway database [16]; (2) the hypergeometric distribution was employed to calculate the probability of a particular group of genes annotated to the pathway, comparing to all the other human genes in the genome; (3) raw *p* value was adjusted for multiple testing using the Bonferroni correction method [17]; (4) pathways with adjusted *p* value < 0.05 were regarded as the significantly enriched pathways.

### Collection of DSCR9 transcription data in human brain

DSCR9 expression levels in different human tissues were collected from the Nonhuman Primate Reference Transcriptome Resource project [18]. The DSCR9 RNA levels in various regions of human brain were obtained from Babru Samal’s molecular brain project (www.molecularbrain.org/). Data on the transcription factor binding sites were collected from Encyclopedia of DNA Elements (ENCODE) project.

### Cell culture

A549 and U231 cells were purchased from American Type Culture Collection. All cells were maintained in DMEM medium (Gibco) supplemented with 10% fetal bovine serum (FBS, Gibco), 100 U/ml penicillin sodium, and 100 mg/ml streptomycin sulfate at 37 °C. All cell lines were passaged for less than 6 months.

### Plasmid construction and stable cell line construction

The genomic segment corresponding to DSCR9 was amplified from human genomic DNA and then cloned into the pcDNA3.1 vector. The correct amplified fragments were identified by restriction endonuclease digestion and were confirmed by sequencing.

Glioma cells U251 and lung cancer cells A549 and were transiently transfected with 4 μg empty vector (pcDNA3.1) as a control or recombinant expression plasmid pcDNA3.1-DSCR9 using Lipofectamine 2000 (Invitrogen, Carlsbad, CA, USA) reagent according to manufacturer’s instructions. The expression of DSCR9 and other predicted genes were determined by qPCR assay at 48 h post transfection.

### Quantitative analysis of DSCR9 and its potential mRNA targets

Total RNAs were extracted from cultured cell lines using the Trizol RNA Reagent (Invitrogen, Carlsbad CA, USA). The RNA concentration was determined by 260/280 nm absorbances using a Nanodrop Spectrophotometer (ND-100, Thermo, USA). QPCR assays were performed using the K1622 RevertAid First Strand cDNA Synthesis Kit (Thermo Scientific) and GoTaq® qPCR Master Mix (Promega) according to the manufacturer’s instructions in an Applied Biosystems 7500 Fluorescent Quantitative PCR System (Applied Biosystems, Foster City, CA, USA). The reaction mixtures were incubated at 95 °C for 30 s followed by 45 amplification cycles of 95 °C for 5 s and 60 °C for 30 s. GAPDH and U6 were used as endogenous controls for mRNA and DSCR9 expressions, respectively. Expressions were normalized to endogenous controls, and the fold change of gene expression was calculated as 2^−ΔΔCt^. Three independent experiments were each performed in triplicates. The primer sequences were listed in additional Additional file 1: Table S1.

## Results

**Fig. 1:**
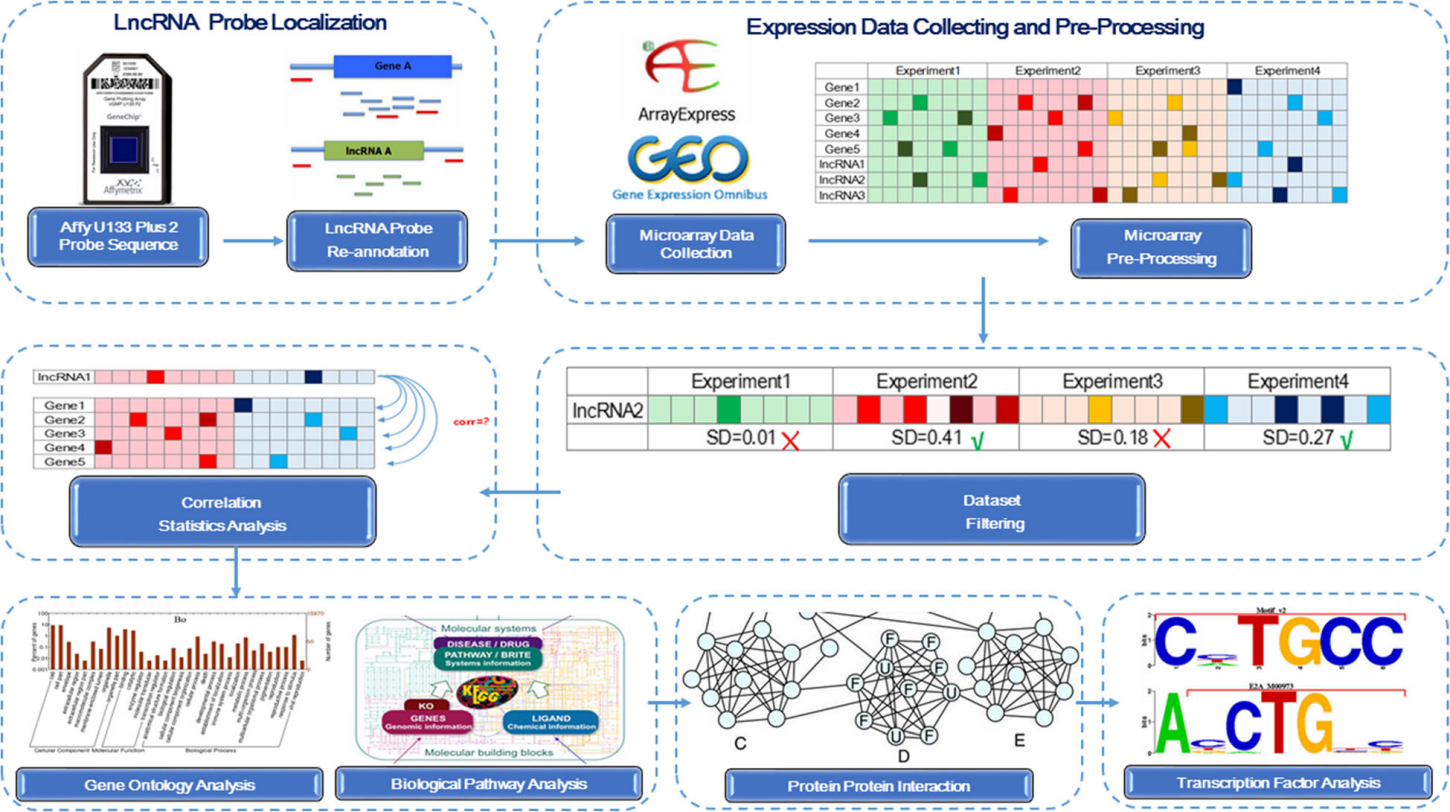
Schematic diagram of the co-expression-interaction network (COIN) Bioinformatics Analysis

To identify the potential target genes of lncRNAs, we developed a bioinformatics analysis. Perl and R scripts mainly create the pipeline that implemented. We obtained nearly 6000 lncRNA probes with high confidence in U133 Plus 2.0 Array. All co-expressed probes showing high correlations with the interesting lncRNA were used for the subsequent gene ontology (GO) analysis, KEGG biological pathway analysis, and protein–protein interaction (PPI) analysis. Additionally, the transcription factor binding sites (TFBS) of our target genes were predicted based on the ENCODE project datasets. The correlation-interaction-network (COIN) bioinformatics analysis was summarized as Fig. 1.

### Validation of COIN prediction with lncRNAs: HOTTIP and HOTAIR

**Fig. 2:**
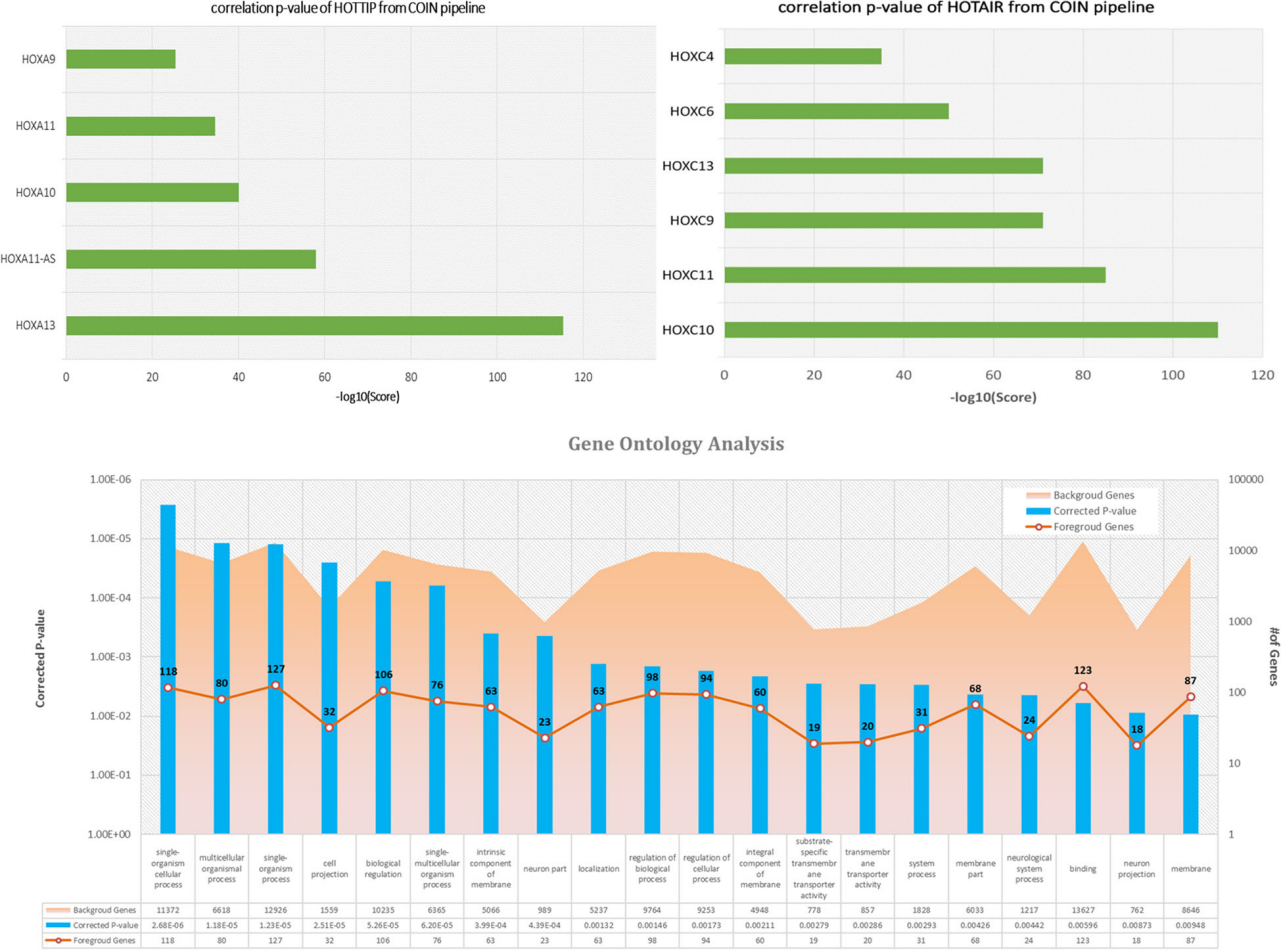
Prediction of lncRNAs HOTTIP and HOTAIR top co-expression genes and functional enrichment pathways by our COIN analysis pipeline

By applying the analysis approach, we found HOTTIP was highly correlated with six HOXA family genes (HOXA13, HOXA11-AS, HOXA10, HOXA11, HOXA9, and HOXA10-AS) that are adjacent to HOTTIP, suggesting the potential regulatory roles of HOTTIP on the HOXA locus genes (Fig. 2). Our HOTTIP target genes and functional analysis was consistent with previous studies, showing that HOTTIP coordinates the activation of several 5’ HOXA genes in vivo, and is involved in forelimb morphogenesis as well as proximal/distal pattern formation [19].

Moreover, we were able to show the potential of the COIN method in predicting the regulated targets of HOTAIR lncRNA (Fig. 2). Our analysis indicated that HOTAIR was highly correlated with six regulated HOXC cluster genes (HOXC10, HOXC9, HOXC6 HOXC11, HOXC13, and HOXC4), and its top pathway functions were embryonic morphogenesis, embryo development, and skeletal system development. Our prediction could be further assessed by the experimental data that was previously reported in Rinn et al. [20].

### DSCR9 functions that predicted by COIN procedure

**Fig. 3:**
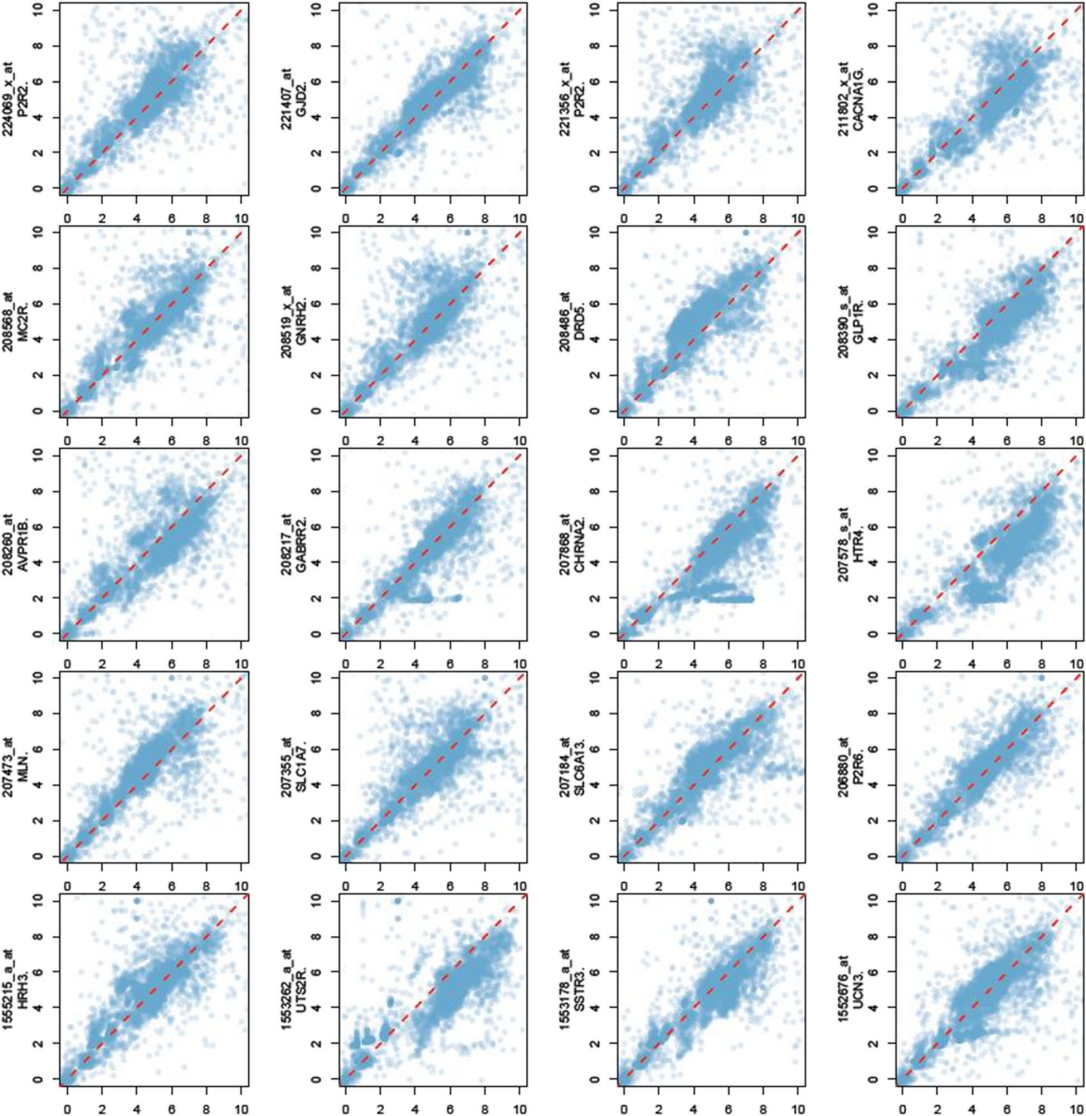
Correlation analysis between DSCR9 probes and top 20 co-expressed genes in HG U133 Plus 2.0 microarray. *x* axis: DSCR9 expression level in microarray experiments; *y*-axis: expression level of gene (with probeset ID) in the corresponding microarray; red dash line represented correlation of 1.0; blue dot represented the expression level of DSCR9-gene pairs in the same microarray

Searching for the most related targets for an lncRNA transcribed from the DSCR on human chromosome 21, and predicting its biological functions with COIN process, we started by calculating the standard deviation of DSCR9 expression level in each dataset. Those with small standard deviation values (< 0.25) were neglected and only 258 sets of experimental data were used in our analysis. Based on Pearson correlation, top 20 DSCR9 co-expression probes were showed in Fig. 3.

**Fig. 4:**
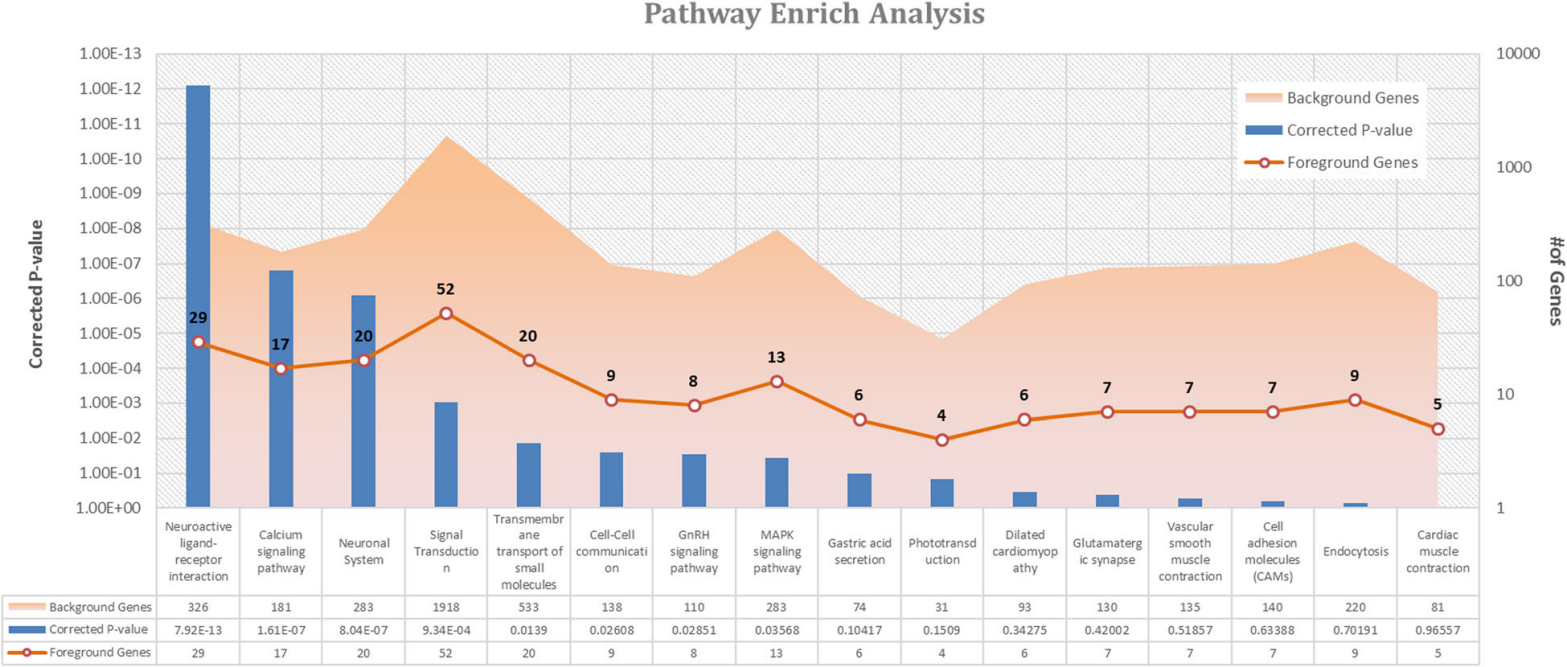
Pathway enrichment analysis of top 1000 co-expression genes. *Blue bars* represented the enrichment significance levels of each pathway. The *orange* empty circle points represented the number of co-expression genes in the corresponding pathway. The area colored in *light orange* represented the number of genes involved

From the list of top 1000 co-expression genes of DSCR9, we conducted biological pathway analysis. There are four pathways that were significantly enriched with these co-expression genes (*p* value < 0.01). The most significant pathways were neuroactive ligand-receptor interaction, calcium signaling pathway, neuronal system, and signal transduction (Fig. 4). Genes that are related to the three most significantly enriched pathways were presented in Table 1.

**Table 1:**
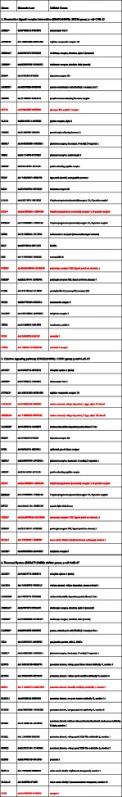
DSCR9 co-expressed genes were related to nervous system by the pathway enrichment analysis

*Genes functioned in more than one neuro-related pathways are bold-faced. Ten genes (P2RX2, SLC8A3, HTR4, CACNG4, UCN3, SYN1, GLP1R, KCNJ5, CACNA1F, and UTS2R) validated by qPCR in two cell lines were highlighted in red

**Fig. 5:**
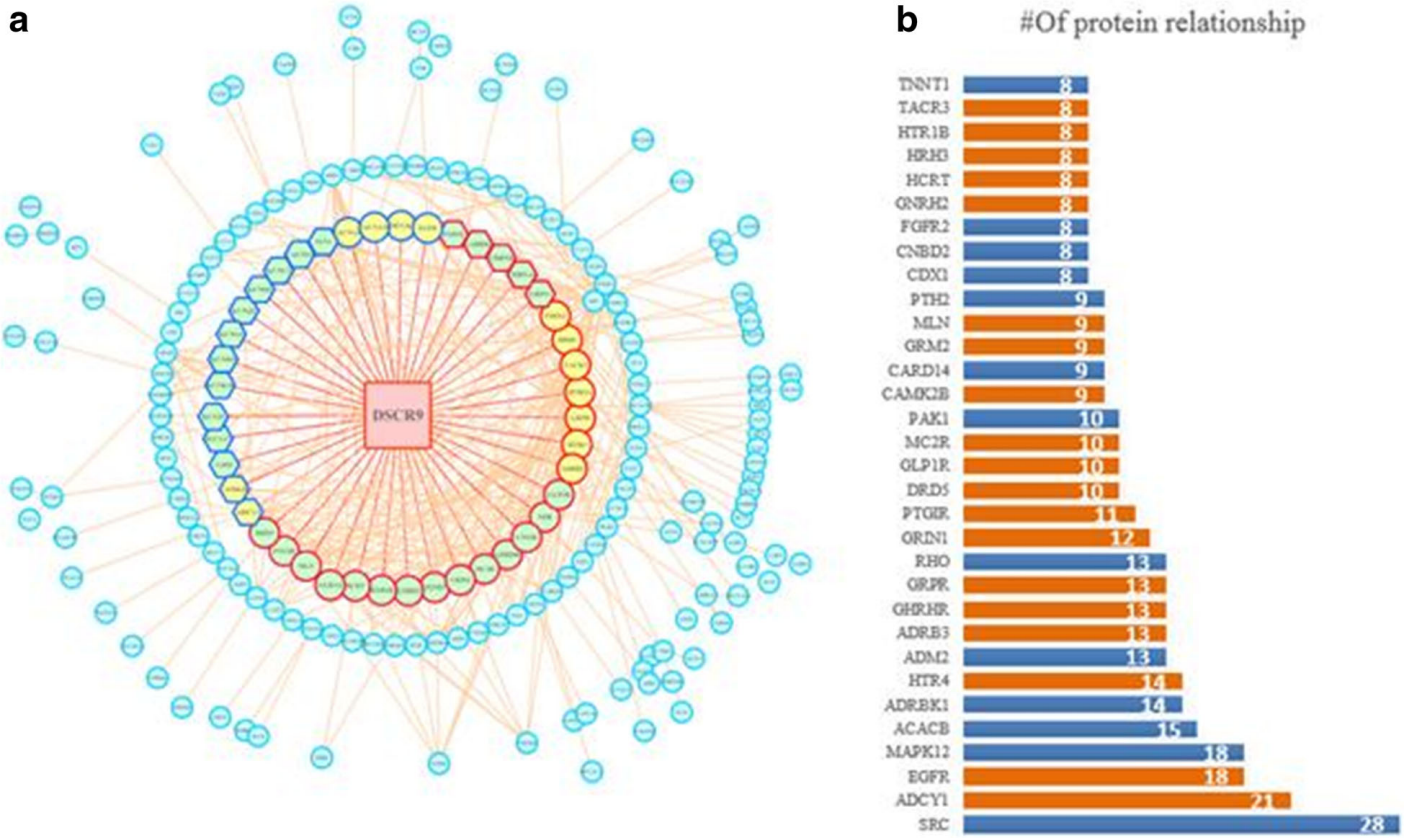
Protein–protein interaction (PPI) network of DSCR9 co-expression genes. **a** DSCR9 Network structure visualized with Cytoscape. Each node represented one gene. Nodes with red border represented co-expressed genes involved in neuroactive ligand-receptor interaction pathway. Nodes filled with light yellow color represented co-expressed genes functioned in calcium signaling pathway, while nodes shaped in hexagonal represent co-expressed genes related to neuronal system. Orange lines show PPI between those highly correlated co-expressed genes of DSCR9. Red lines represented potential relationships between DSCR9 and its targets. **b** The core DSCR targeted genes in the PPI network were listed with their gene symbols and weights. Numbers in the bars showed the interaction weight of the corresponding genes in DSCR9 network (Fig. 4a). Orange-colored bars indicated that the corresponding genes were members of the neuro-related pathways

Moreover, we performed PPI analysis to evaluate the interacting relations of these potential DSCR9 targets with Cytoscape (Fig. 5a). lncRNA DSCR9 exhibited strong interactions with those neuro-related genes (nodes presented in the inner circle). For a further data-mining of this network, we calculated the interaction weight (numbers of neighbors) of each core node (Fig. 5b). Consistently, most of the core genes in the identified PPI network were neuro-related.

### DSCR9 expression in human brain tissues

**Fig. 6:**
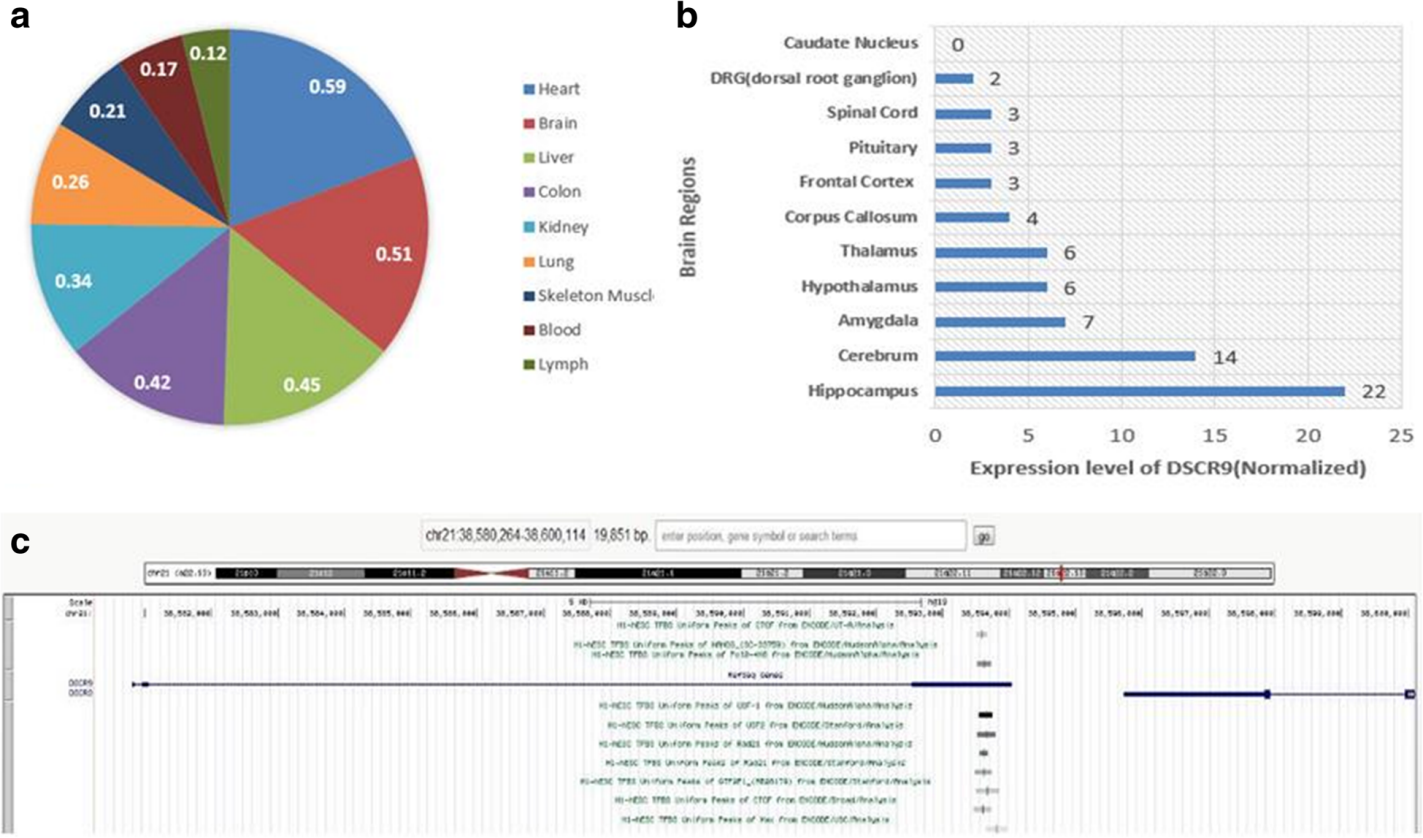
Expression and regulation of DSCR9 in human tissues and brain regions. **a** DSCR9 expression levels (shown by FPKM levels, fragment per kilometer) in a series of human tissues (data obtained from NHPRTR project). **b** DSCR9 expression levels in different brain regions (data obtained from molecularbrain.org). **c** DSCR9 with four transcription-factor-binding-sites (TFBS) in the third DSCR9 exon displayed with UCSC genome browser

For in-depth examination on DSCR9 expression, we collected data from three international projects and performed integrative analysis of the transcription level of DSCR9. The expression of DSCR9 lncRNA was tissue-specific. Among nine different human tissues, DSCR9 displayed the highest abundance in hearts and brains (Fig. 6a).

The lncRNA levels were also analyzed in various regions of the human brain, and the highest DSCR9 RNA level was found in the hippocampus followed by those in the cerebrum, amygdala, etc. (Fig. 6b). Transcription factor binding site (TFBS) data obtained from ENCODE project was analyzed. Four TFBSs in the third exon of DSCR9 and three transcription factors including USF1, USF2, and Rad21 might be involved in its expression regulation via these binding sites (Fig. 6c).

### Genes that relate to DSCR9 co-expression oncology

Using the COIN analysis, we found 14 genes that functioned in more than one neuro-related pathway, including EGFR (Table 1). EGFR was also abundantly detected in the brain by Nieto-Estevez et al. [21]. It was critical in neurological processes including cell death, the survival and differentiation of neurons. EGFR gene has been reported to be dysregulated in the mouse model of Down syndrome (DS), with regulatory impacts on calcium signaling pathway, neuroactive ligand-receptor interaction, and the MAPK signaling pathway [22]. Our analysis results are concordant with the abovementioned reports.

Patients with DS exhibited considerably reduced incidence of most solid tumors [23, 24], and their overall cancer mortality rate was approximately 10% below the normal level of general population. Although the cause was still unclear, it had been proposed that the DS patients might get extra doses of one or multiple cancer-suppressor genes such as DSCR1 and DYRK1A due to the extra copy of chromosome 21 [25]. An extra copy of DSCR1 was sufficient to significantly suppress angiogenesis and tumor growth and its protein, DSCR1, was upregulated in tissues from people with DS [25]. The attenuation of calcineurin activity by DSCR1 together with another chromosome 21 gene DYRK1A, might be sufficient to remarkably diminish angiogenesis. No direct evidence had been reported so far to illustrate that DSCR9 was responsible for cancer progressing. However, it seemed to be a reasonable hypothesis from our analysis.

The proposed COIN approach showed that SRC gene was the most outstanding target candidate in the regulatory network of DSCR9 (Fig. 5b). SRC gene was reported to encode a proto-oncogene tyrosine-protein kinase. Activation of the Src pathway had been observed in about 50% of tumors from colon, liver, lung, breast, and the pancreas [26]. Another significant regulatory target of DSCR9 in the identified network was EGFR, which was a well-studied oncogene. It has been identified as an important drug target and understanding this gene has led to the development of multiple anti-cancer therapeutics (known as ‘EGFR inhibitors’) such as gefitinib, erlotinib, afatinib, brigatinib and icotinib for lung cancer, and cetuximab for colon cancer. Additionally, gastrin-releasing peptide receptor (GRPR) was also identified as an important target candidate of DSCR9, and diseases that associated with GRPR include lung cancer and prostate adenocarcinoma. In summary, the COIN analysis results indicated that DSCR9 was highly correlated with several oncogenes and therefore it was very likely that its regulatory function could be employed to explain the reduced cancer incidence in patients with DS.

### DSCR9 co-expression genes that validated in cell lines

**Fig. 7:**
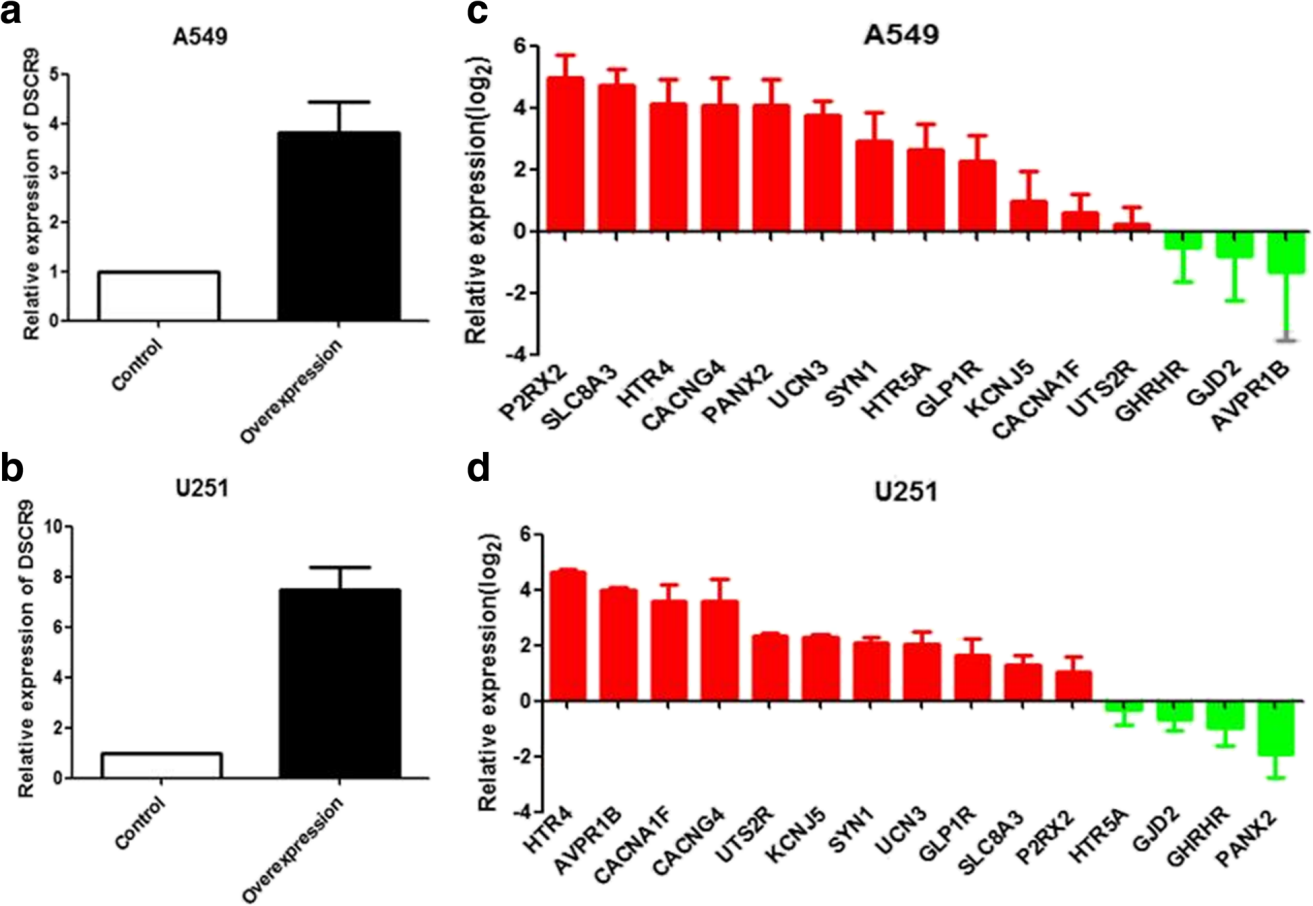
Bioinformatics predictions were validated by QPCR. **a** The DSCR9 stable overexpression A549 and U251 cell lines were constructed. **b** QPCR analysis showing the predicted co-expressed genes were upregulated in both A549 and U251 upon DSCR9 overexpression

To further examine the predictive capability of the COIN model, a standard gene expression analysis was performed in two DSCR9 over-expressed cell lines. After the construction and validation of DSCR9-overexpression in A549 and U251 cells (Fig. 7a–b), we selected 15 top DSCR9-associated genes in our prediction and determined their expression levels by qPCR. We found that 12 of these 15 predicted co-expressed genes were unregulated upon DSCR9 over-expression in A549 cells, and 11 of them were upregulated in U251 (Fig. 7c–d). In conclusion, our experimental data showed high consistency (> 73%) with the bioinformatics analysis predictions, supporting the reliability of the proposed COIN method.

## Discussion

Although the dysregulation of some lncRNAs had been linked to human diseases, the underlying molecular mechanisms, including the regulatory function of many lncRNAs have not been fully elucidated. An effective approach is in need to provide a feasible guidance for functional studies of these non-coding RNAs. COIN method aimed for the prediction of targeted lncRNA function and its regulatory network. We validated the COIN approach by predicting the interaction networks of two well-known lncRNAs: HOTAIR and HOTTIP. Moreover, reliability of its predictions was also confirmed by a quantitative PCR in two independent DSCR9 over-expression cell lines.

### Potential neurological pathway genes that relate DSCR9 to DS

DS is a neurobehavioral specificity disease [27]. Three pathways that are significantly enriched with DSCR9 co-expression genes associated with neurological functions, with top 10 qPCR validation genes in parentheses (see Fig. 4 and Table 1):

In the COIN analysis, we found 14 genes that functioned in more than one neuro-related pathway, including HTR4, P2RX2, and ADCY1 (Table 1). ADCY1 was found to encode a form of adenylate cyclase that is expressed in the brain and was involved in regulatory processes in the central nervous system. Previous studies indicated that it might play significant roles in memory acquisition and learning. Overall, combining the COIN analysis results and literature reports, we believe that the three pathways in which the DSCR9 co-expression genes were enriched are essential for the nervous system development. HTR4 is another gene induced by DSCR9 over-expression. HTR4 is a glycosylated transmembrane protein that functions in both the peripheral and central nervous system to modulate the release of various neurotransmitters. Previous studies indicated that HTR4 might be associated with neurological diseases such as Parkinson’s disease 5 [28, 29]. The product of P2RX2 gene belongs to the family of purinoceptors for ATP. This receptor functions as a ligand-gated ion channel. Binding to ATP mediates synaptic transmission between neurons and from neurons to smooth muscle [28, 29].

Components of the neuro-active ligand-receptor interaction pathway showed high relevance to DS acute lymphoblastic leukemia [30]. Furthermore, it had been well studied that calcium ions generated versatile intracellular signals, supporting that calcium signaling pathway also played an important role in all types of neurons [31]. Impaired calcineurin activity was already linked to many human diseases including DS, Alzheimer’s disease, brain ischemia, cardiac hypertrophy, and maybe more [32]. Interestingly, DSCR1 gene was upregulated in DS patients and encoded a protein that suppressed VEGF-mediated angiogenic signaling by the calcineurin pathway [25]. Previous report also suggested that presynaptic calcium channels might serve as the regulatory node in a dynamic, multilayered signaling network that exerted short-term control of neurotransmission in response to synaptic activity [33]. A transient rise of the calcium level in dendritic spines was essential for the induction of activity-dependent synaptic plasticity.

In addition, our experimental data supported the predicted DSCR9 regulation of its target genes in two different cancer cell lines, i.e., A549 and U251. Over 73% of our predicted DSCR9 target genes were upregulated when DSCR9 was over-expressed. Consistent with our gene ontology (GO) analysis, two of the unregulated genes, say, CACNG4 [34] and CACNA1F [35], encoded the subunits of calcium channels, which were involved in the calcium signaling pathway.

According to the co-expressed genes of DSCR9 in the resulting network, we predicted that DSCR9 might also contribute to the cardiovascular- and cerebral-related diseases. Consistently, data from NHPRTR project indicated that the highest expression levels of this lncRNA were detected in heart and brain (Fig. 6a). Moreover, compared to other regions of the brain, the abundance of DSCR9 seemed to accumulate in hippocampus (Fig. 6b). This supported our prediction that this lncRNA might be a factor leading to abnormal dendritic branching and spine number, and further reduction of brain volume as it had been previously reported that a reduction in brain volume in patients with DS was attributed to impaired dendritic and synaptic maturation [36]. Dendritic branching and spine number were dramatically reduced in pyramidal neurons in the hippocampus, visual cortex, and motor cortex after 4 months postnatal age in individuals with DS [28, 29].

### Potential transcription factors that relate DSCR9 to DS

In the COIN analysis of DSCR9, we identified three transcription factors including Rad21, USF1, and USF2 that were highly correlated with DSCR9 (Fig. 6c), and we further noticed that putative binding sites of these transcription factors were present in the third exon of DSCR9. Intriguingly, these transcription factors had been shown to be related to DS. Rad21 gene was found to be highly mutated in DS [37], and its expression level was elevated in DS patients [38]. Similarly, USF1 expression level was positively related with dCK gene [39], which was a well-documented DS leukemia-related gene [40]. Moreover, previous reports suggested that USF1 played a trans-activating role on the CBS-1b promoter [41], and CBS-1b gene had been accepted as a DS risk factor [42, 43]. The third transcription factor that is related to DSCR9 was USF2, whose expression was shown to be increasing during aging [44]. Interestingly, DS patients exhibited an increased risk of many chronic diseases, which were typically associated with aging. Previous reports suggested that trisomy 21 was linked to clinical manifestations of accelerated aging, and DSCR9 appeared to be a negative epigenetic clock controlling tissue aging in the brain [45]. Supporting its potential activities and expression regulation in DS, it was also found that DSCR9 displayed abnormal methylation pattern in DS patients [46], and the methylated sites were mainly located in the third exon [47]. All together, these results implicated a DS-related transcription regulation of DSCR9 lncRNA, which may involve altered DNA methylation patterns and/or chromatin structure as well as the transcription factors USF1/USF2/Rad21 binding to the exon 3 of DSCR9.

## Conclusions

In conclusions, our studies established a solid bioinformatics pipeline for functional predictions of DS transcriptome associations. Our qPCR assay showed that the expression of these genes were induced by DSCR9, implying that these genes might be regulated by DSCR9. The results provided a valuable guidance for further investigations on the regulatory mechanism of DSCR9 as well as its relevance to DS and other neurological diseases. Dysregulation of DSCR9 and/or its target genes in these pathways might be responsible for the pathogenesis and progressing of DS. As extension of this work, generalized approach can be adapted for other disease-related transcriptome association studies.

## Additional file

Additional file 1: Table S1. Primers used in this study. (DOCX 15 kb)

## Abbreviations

COIN: Correlation-interaction-network
DS: Down syndrome
DSCR: Down syndrome critical region
lncRNA: Long non-coding RNA
PPI: Protein–protein interaction
