# Supplementary material for: Identify Down syndrome transcriptome associations using integrative analysis of microarray database and correlation-interaction network
Source: Hum Genomics. 2018 Jan 19;12:2. doi: 10.1186/s40246-018-0133-y (PMC5775600; doi:10.1186/s40246-018-0133-y)
Supplement: Additional file 1: Table S1. — Primers used in this study. (DOCX 15 kb) [file 40246_2018_133_MOESM1_ESM.docx]

# Table S1. Primers used in qPCR validation of DSCR9 co-expression genes

| Gene | Sense (5’-3’) | Antisense(3’-5’) |
| --- | --- | --- |
| DSCR9 | cccatggcaactgtgctgg | cacaggttattagctgcgtcacca |
| RNCU6 | ggaacgcttcacgaatttgcg | tgctcgcttcggcagcac |
| ACTN1 | ttcaagatcctggctgggg | cagtactcagcctggtcggg |
| AVPR1B | CCTCTGAACGATTTCCGCCT | AAAATGGAGTGGCAGGGGAG |
| CACNG4 | AAATCAACGCCAGGAGAGCA | CAGCTCGGTCCGTTTATCCA |
| CACNA1F | CCAAAGCTGGGGGAAGAGAG | GCTCTGGGGTGGTGTCTTTC |
| GLP1R | GTCCAAGCGAGGGGAAAGAA | ATCGCAGAGGCGATAACCAG |
| GHRHR | GCCCGTTACCGACCGTATT | AGACAGGCACTCTCATCCTCT |
| HTR4 | GTGAGATCGCTCCAGCCC | GACTCCACGGGCTCAACA |
| HTR5A | AACGGATGCTCACTAGCAGG | TTGCAGAAGGGGTGTTCAGG |
| GJD2 | GAGCTGACTCCACACCCATC | TTCCAGGGCATTTCGGAACA |
| KCNJ5 | ACAGGGAGAGGTTCCAGCTAC | TGATGCTAACTTCTGGGGTGT |
| P2RX2 | GAGCTCGCACACAAGGCA | TTGGGGTTGCACTCCGATG |
| PANX2 | CACATCAACACGCTATCCTCA | ACGGTGCCATCCCTCAAAAC |
| SLC8A3 | GTCTGAGTATCAACTCTTCCAGCA | ACTTATCCACCCTCCTGGCT |
| SYN1 | CTGACCAATGCCTTCAACCTTC | GAGTGGGGTATCAGTCGGAG |
| UCN3 | TATACACACAGGGGAGGGGA | CATGAACTTGTGGCTGCTCC |
| UTS2R | TCGTGAGGGGTCAGAGATGG | GCTGTTGAGGGTTGCGTTG |
